# Association Between Sleep and Cardiovascular‐Kidney‐Metabolic Syndrome: Mediation Analysis of Inflammatory Biomarkers

**DOI:** 10.1155/mi/9072641

**Published:** 2026-04-24

**Authors:** Minjie Hu, Ying Xu, Jiao Ming, Xuelin Zhang, Xiaolu Bian, Xinrui Liang, Haiyan Wang, Longyi Zheng, Ying Zhang, Zhiyong Guo

**Affiliations:** ^1^ Department of Nephrology, First Affiliated Hospital of Naval Medical University, Shanghai Changhai Hospital, Shanghai, China, chhospital.com.cn; ^2^ Department of Nephrology, Huadong Hospital Affiliated to Fudan University, Shanghai, China, fudan.edu.cn; ^3^ Department of Endocrinology, First Affiliated Hospital of Naval Medical University, Shanghai Changhai Hospital, Shanghai, China, chhospital.com.cn; ^4^ Department of Clinical Pharmacy, General Hospital of Central Theater Command, Wuhan City, Hubei Province, China

**Keywords:** cardiovascular-kidney-metabolic syndrome, sleep pattern, systemic immune inflammation index, systemic immune response index

## Abstract

**Background and Aims:**

Sleep disturbances are linked to individual cardiometabolic diseases, but their association with the emerging cardiovascular‐kidney‐metabolic (CKM) syndrome, a constellation of interrelated conditions, remains less clear. To evaluate the association of sleep factors (sleep duration, diagnosed sleep disorder, self‐reported trouble sleeping) with the risk of CKM syndrome.

**Methods and Results:**

We included 11,949 adults from the National Health and Nutrition Examination Survey (NHANES). A composite sleep score (0–3, from healthy to poor) was derived from sleep duration, diagnosed sleep disorder, and self‐reported trouble sleeping. CKM syndrome was defined and staged (Stage 0–4) based on the coexistence and severity of cardiometabolic and kidney diseases. Systemic inflammation was assessed using the systemic immune‐inflammation index (SII) and systemic inflammation response index (SIRI). A poor sleep pattern was significantly associated with higher odds of CKM syndrome (fully adjusted odds ratio [OR] = 1.64, 95% confidence interval (CI): 1.37–1.95) and with more advanced CKM stages in an ordinal model, indicating a dose–response relationship. In exploratory mediation analysis, SII and SIRI appeared to partially mediate the association between self‐reported trouble sleeping and CKM syndrome in initial models. However, this mediating effect was substantially attenuated and became nonsignificant for SII after comprehensive adjustment for adiposity (body mass index (BMI) and waist circumference) and lifestyle factors.

**Conclusions:**

Poor sleep patterns are robustly associated with an increased risk and severity of CKM syndrome. While systemic inflammation was correlated with both sleep disturbances and CKM, its role as an independent mediator was largely explained by shared variance with obesity and metabolic factors. These findings underscore sleep health as a potential target for multimodal risk reduction within the CKM framework, though causality needs confirmation in longitudinal studies.

## 1. Introduction

Chronic kidney‐metabolic syndrome, characterized by the coexistence of cardiovascular disease (CVD), chronic kidney disease (CKD), and metabolic disorders, poses a significant health burden on patients and society at large. CKM syndrome is a health disorder described in a consensus statement by the American Heart Association (AHA) in 2023, aiming to explore the interconnections among cardiovascular, kidney, and metabolic (CKM) conditions, and subsequently, develop relevant measures to reduce morbidity and mortality [1]. These conditions are leading contributors to morbidity and mortality, resulting in substantial economic costs associated with their management and treatment [2, 3]. According to the scientific statement from the AHA [1], CKM syndrome was classified into distinct stages, ranging from Stages 0 to 4. In Stage 0, individuals currently do not have clearly defined risks for CVD, kidney disease, or metabolic disease. Individuals in Stage 1 present risk factors such as excess body fat, unhealthy fat distribution, impaired glucose tolerance, or prediabetes. And they need to adopt a balanced diet and engage in regular physical exercise to alleviate these risk factors. In Stage 2, individuals begin to develop type 2 diabetes, high blood pressure, elevated triglycerides, or kidney disease, indicating an increased risk of deterioration in heart and kidney health. Stage 3 includes individuals with metabolic risk factors, kidney disease, or a high predicted risk of CVD, who are at risk of developing early CVD. Stage 4 CKM was defined as clinical CVD and further subdivided into two groups: Stage 4a, comprising individuals with clinical CVD but without kidney failure, and Stage 4b, including those with both clinical CVD and kidney failure.

The occurrence of CKM health issues within the population is substantial. In the United States, the prevalence rates for heart, kidney, and metabolic diseases are ~9% to 11%, 15%, and 13%, respectively [4, 5]. In China, recent data indicates that the prevalence rates for CVD, type 2 diabetes, and CKD are 23.4%, 11.2%, and 10.8%, respectively [6–8]. The prevalence of CKM syndrome underscores the need for reliable biomarkers to facilitate early detection and effective management. Sleep is a vital physiological process that plays a crucial role in maintaining overall health and well‐being. It has been extensively studied for its impact on various biological functions, including metabolic regulation [9], cardiovascular health [10, 11], and immune response. Sleep deprivation or poor sleep quality has been linked to a range of health issues, particularly CVDs and metabolic disorders [12], which are key components of CKM syndrome. The underlying mechanisms by which sleep influences these conditions are multifaceted, involving hormonal regulation, inflammatory pathways, and metabolic homeostasis. However, the interplay between lifestyle factors, such as sleep patterns, and the development of CKM syndrome remains poorly understood. This underscores the necessity for further research to elucidate the relationships between sleep quality and CKM syndrome, which may ultimately inform more effective prevention and management strategies. Therefore, we hypothesize that systemic inflammatory indicators such as the systemic immune inflammation index (SII) or systemic inflammation response index (SIRI) mediate the relationship between sleep patterns and CKM syndrome.

The SII and the SIRI are two new composite indices that combine three distinct white blood cell subsets along with platelets, these indices reflect the interplay between thrombocytosis, inflammation, and immune response. Furthermore, these indices have been extensively utilized in research to evaluate the relationship between chronic inflammatory status and a range of human diseases, such as the risks of heart failure (HF) [13], CVD and all‐cause mortality [14], CKD [15], metabolic disorders [16], and inflammatory conditions. Collectively, based on the importance of inflammatory biomarkers for CVD, CKD, and metabolic disorders, respectively, we hypothesized that sleep disturbances may increase CKM risk, in part, by promoting a state of chronic systemic inflammation. To verify this, we employed the SII and SIRI as integrative biomarkers that capture the balance between pro‐inflammatory neutrophils, adaptive immune lymphocytes, and thrombotic potential (platelets), which have been implicated in the pathogenesis of cardiometabolic and kidney diseases.

As noted above, we conducted a cross‐sectional study to explore the relationship between sleep and CKM syndrome using data from the National Health and Nutrition Examination Survey (NHANES), with a particular focus on the role of inflammatory biomarkers as potential mediators. Also, our study is the first population‐based mediation analysis that connects sleep patterns to a combined CKM outcome using SII/SIRI.

## 2. Methods

### 2.1. Study Population

We designed a cross‐sectional study using data from the NHANES between 2007 and 2018. We designed a cross‐sectional study using data from the NHANES for 12 consecutive years: 2007–2008, 2009–2010, 2011–2012, 2013–2014, 2015−2016, and 2017–2018. In this article, we weighted the data according to the sample weight calculation method recommended by NHANES, combining the data for 12 years from 2007 to 2018, with the 12‐year weight equal to 1/6 of the 2‐year weight fasting subsample weight (WTSAF2YR) by six (Tables S1 and S2).

The survey protocol was approved by the NCHS Institutional Review Board, and all participants provided written informed consent. The NHANES data used for this analysis is available at https://www.cdc.gov/nchs/nhanes. Initially, 59,842 participants were included in the study, we employed a set of exclusion criteria, and meeting any one of the following will result in exclusion from our research: (1) below the age of 20 and pregnant women; (2) those with incomplete data necessary for diagnosing CKM syndrome; (3) those with incomplete data in the SII and SIRI calculations; (4) those with no sample weights; (5) those with incomplete data on significant covariates. Finally, a total of 11,949 participants were enrolled in the analysis (Figure S1).

### 2.2. Definition of CKM Syndrome

CKM syndrome is the coexistence of subclinical or clinical CVD, CKD, and metabolic disorders. CVD was defined as any of HF, coronary heart disease, heart attack, or stroke. Subclinical CVD was defined as having ≥20% of 10‐year CVD risk or high‐risk CKD [17]. Following the Kidney Disease: Improving Global Outcomes (KDIGO) classification, we categorized CKD risk based on estimated glomerular filtration rate (eGFR) thresholds (<30, 30–44, 45–59, and ≥60 mL/min/1.73 m^2^) and urinary albumin‐to‐creatinine ratio (UACR) levels (<30, 30–299, and ≥300 mg/g) (Table S3), and CKD was defined as moderate‐ or high‐risk levels of CKD in our study [18]. Metabolic syndrome was defined by the presence of at least three of the following five criteria [19]: (1) waist circumference ≥102 cm for men or ≥88 cm for women; (2) serum TG ≥150 mg/dL; (3) Low HDL cholesterol: serum HDL‐c <40 mg/dL for men or <50 mg/dL for women; (4) systolic blood pressure (SBP) ≥130 mmHg or diastolic blood pressure (DBP) ≥85 mmHg, or undergoing antihypertensive treatment; (5) fasting plasma glucose ≥100 mg/dL or undergoing antidiabetic treatment.

We classified participants by four CKM stages according to the different clinical severities of CKM syndrome: Stage 0 was defined as individuals with normal body mass index (BMI <25 kg/m^2^) and waist circumference (<88 and <102 cm for women and men, respectively), normoglycemia, normotension, a normal lipid profile, and with no evidence of CKD or subclinical or clinical CVD; Stage 1 was defined as individuals with overweight/obesity (BMI ≥25 kg/m^2^), abdominal obesity (waist circumference ≥88/102 cm in women/men), or dysfunctional adipose tissue, without the presence of other metabolic risk factors or CKD; Stage 2 was defined as individuals with at least 1 of the other metabolic disorders (hypertriglyceridemia, hypertension, metabolic syndrome, and diabetes) or CKD; Stage 3 included individuals with subclinical CVD or with very high‐risk CKD, Subclinical CVD was defined as either a predicted 10‐year CVD risk of 20% higher or the presence of high‐risk CKD. The 10‐year CVD risk was estimated using the simplified CKM risk algorithm, which includes factors such as age, sex, smoking status, blood pressure, cholesterol levels, diabetes status, kidney function, and the use of antihypertensive and statin medications [20]; Stage 4 was defined as clinical CVD, including coronary heart disease, congestive HF, and stroke among individuals with metabolic disorders or CKD.

### 2.3. Measurements of Sleep Factors and Definition of a Sleep Pattern

Sleep duration was assessed by the question “How much sleep do you usually get at night on weekdays or workdays?” Referring to the recommendations of the National Sleep Foundation’s sleep duration [21], the categorical variable of sleep duration was divided into three groups: short (<7 h/day), normal (7–9 h/day), and long sleep time (>9 h/day), and 7–9 h per night was used as the reference group. Sleep disorder and trouble sleeping were measured according to the response to the questions “Have you/has SP ever been told by a doctor or other health professional that you have a sleep disorder?” and “Have you ever told a doctor or other health professional that you have trouble sleeping?”, respectively. For each sleep factor (sleep duration, self‐reported trouble sleeping, and sleep disorder), lower risks were assigned a value of 1 and higher risks a value of 0 to calculate overall sleep scores, which could range from 0 to 3. A sleep score of 0–1, 2, or 3 corresponded to a poor, intermediate, or healthy sleep pattern, respectively [22].

### 2.4. Measurements of Inflammatory Biomarkers

In this study, the inflammatory biomarkers of the subjects participating in the examination were SII and SIRI. The platelet count (PC), the neutrophil count (NC), the monocyte count (MC), and the lymphocyte count (LC) were measured separately, with units of 1000 cells/µL [1]. Their calculation results are as follows:

SII=Platelet count×neutrophil count/lymphocyte count,

SIRI=Neutrophil count×monocyte count/lymphocyte count.

To comprehensively evaluate the mediation of inflammation index between sleep and CKM syndrome, participants were equally classified into four groups according to SII or SIRI distribution: lower SII (Q1, <311.17) or SIRI (Q1, <0.64), low middle SII (Q2, 311.17–434.75) or SIRI (Q2, 0.64–0.94), middle SII (Q3, 434.75–619.88) or SIRI (Q3, 0.94–1.40) and high SII (Q4, >619.88) or SIRI (Q4, >1.40).

### 2.5. Assessments of Other Covariates

We ultimately investigated the inclusion of relevant potential confounding factors, including demographic information: age (years), sex (male, female), race/ethnicity (Mexican American, Other Hispanic, Non‐Hispanic White, Non‐Hispanic Black, Other Race), education level (≤high school, high school, ≥high school). Smoking status (never, former, and now) and alcohol drinking (never, mild, moderate, and heavy drinkers) were obtained from the cigarette use and alcohol use questionnaires, respectively. BMI was calculated as weight (kg) divided by height squared (kg/m^2^). The sedentary time (<5 h, ≥5 h) was referred to as the duration spent sitting in a typical day, excluding sleeping. Owing to the use of weighted logistic regression analysis, our study categorized CKM syndrome into two groups for ease of analysis: one group includes CKM Stages 0–2, while the other group consists of CKM Stages 3–4. To assess potential multicollinearity among covariates included in the multivariable regression models, we calculated the variance inflation factor (VIF) for each covariate. A VIF value greater than 10 was considered indicative of problematic multicollinearity. The results of the multicollinearity analysis are presented in Table S4.

### 2.6. Statistical Analysis

According to the analysis guidelines published by the National Center for Health Statistics, stratification and primary sampling units were considered for complex, multistage, probability sampling designs. To account for unequal sampling probabilities and nonresponses, the data from all participants were weighted based on the NHANES examination weights and the fasting subsample weights. Continuous variables were presented as weighted mean ± standard error (SE) for normally distributed data and median (interquartile range [IQR]) for skewed data. Categorical variables were presented as frequencies (weighted percentages) and compared using design‐based chi‐square tests.

To assess potential selection bias due to missing data, we compared the baseline characteristics of included participants with those excluded from the analysis among individuals aged ≥20 years. Continuous variables were presented as median (IQR); categorical variables were presented as number (percentage) and compared using chi‐squared tests. The results of this comparison are presented in Table S5. Sensitivity analyses included subgroup analyses excluding participants with preexisting conditions (Table S6) and a negative control outcome analysis examining the association between sleep patterns and accidental injury (Table S7).

Weighted univariate and multivariate logistic regression was used to calculate the odds ratios (ORs) and 95% confidence intervals (95% CIs) to assess the relationship between each sleep factor and CKM syndrome and its components. Multivariate logistic regression included demographic information and vital factors associated with sleep and CKM syndrome. ORs were calculated through three models: an unadjusted model (Model 1), an age, sex, race/ethnicity, and education level‐adjusted model (Model 2), and Model 3 for potential confounders, including age, sex, race, education level, BMI, waist circumference, smoking status, alcohol drinking, and sedentary time.

Restricted cubic spline (RCS) analysis with four knots was used to explore potential nonlinear relationships between sleep duration and CKM syndrome and its components after adjustment for multiple covariates. Finally, following the recommendations of Tyler VanderWeele [23], mediation analysis was conducted to explore the possible mediating effects of SII and SIRI in the relationship between sleep factors and CKM syndrome. The potential mediating roles of inflammatory biomarkers (SII and SIRI) were assessed through both single and multiple mediation analyses, using the mediation and bruceR R packages, respectively. The proportion mediated was quantified as the indirect effect divided by the total effect. In the single‐mediator analysis, effects are expressed on the probability scale; in the multiple‐mediator analysis, effects are expressed as logistic regression coefficients. The statistical significance of the mediating effects was tested using nonparametric bootstrap sampling with 1000 resamples. All analyses were conducted using R 4.4.1 (R Foundation for Statistical Computing, Vienna, Austria). A two‐sided *p*  < 0.05 was considered significant.

## 3. Results

### 3.1. Baseline Characteristics of Study Participants

The baseline characteristics of the participants according to CKM stages are shown in Table 1. In general, there were significant differences in age, gender, race/ethnicity, education level, smoking status, hypertension, diabetes, CKD, and CVD among different CKM stages (*p*  < 0.001). Compared with those in Stage 0, participants in Stages and 4 were more likely to be male, non‐Hispanic White, former or current smokers, more likely to be obese, have high levels of waist, have poor sleep pattern, and have lower education levels.

**Table 1:** Baseline characteristics of participants according to CKM stages.

*N*	Stage 0	Stage 1	Stage 2	Stage 3	Stage 4	*p*‐Value	SMD
	1109	2168	6333	1031	1308		
Age (median [IQR]), years	31.00 [24.00, 42.00]	40.00 [29.00, 51.00]	48.00 [36.00, 59.00]	77.00 [72.00, 80.00]	66.00 [57.00, 75.08]	<0.001	1.728
Gender (%)						<0.001	0.251
Male	322 (27.7%)	1092 (53.4%)	3278 (53.4%)	582 (51.0%)	756 (56.4%)	—	—
Female	787 (72.3%)	1076 (46.6%)	3055 (46.6%)	449 (49.0%)	552 (43.6%)	—	—
Race/ethnicity (%)						<0.001	0.199
Mexican American	105 (3.8%)	415 (13.2%)	1095 (9.7%)	107 (5.1%)	133 (4.7%)	—	—
Other Hispanic	144 (8.1%)	257 (9.0%)	743 (5.9%)	98 (5.3%)	120 (4.1%)	—	—
Non‐Hispanic White	506 (71.8%)	847 (59.4%)	2435 (65.7%)	539 (72.6%)	690 (72.4%)	—	—
Non‐Hispanic Black	111 (7.7%)	398 (10.8%)	1262 (10.9%)	204 (11.6%)	271 (11.5%)	—	—
Other Race	243 (8.5%)	251 (7.6%)	798 (7.9%)	83 (5.4%)	94 (7.3%)	—	—
Education level (%)						<0.001	0.327
≤High school	139 (16.0%)	434 (16.7%)	1570 (16.3%)	343 (25.1%)	440 (25.1%)	—	—
High school	203 (18.8%)	419 (21.0%)	1481 (23.4%)	274 (29.0%)	322 (26.6%)	—	—
≥High school	767 (65.3%)	1315 (62.3%)	3282 (60.2%)	414 (45.9%)	546 (48.3%)	—	—
BMI (median [IQR]), (kg/m^2^)	22.00 [20.40, 23.40]	27.30 [25.00, 30.60]	29.39 [25.80, 33.90]	28.00 [25.02, 32.20]	29.28 [25.60, 34.00]	<0.001	0.802
Waist (median [IQR]), (cm)	79.72 [74.60, 83.80]	94.70 [88.00, 103.40]	101.70 [92.50, 112.30]	103.40 [94.30, 112.08]	104.96 [95.59, 116.60]	<0.001	1.032
Sleep pattern (%)						<0.001	0.309
Healthy	657 (59.4%)	1169 (56.4%)	2844 (45.0%)	495 (48.4%)	467 (37.6%)	—	—
Intermediate	361 (32.3%)	794 (35.0%)	2450 (38.1%)	378 (35.7%)	451 (32.8%)	—	—
Poor	91 (8.3%)	205 (8.6%)	1039 (16.9%)	158 (15.9%)	390 (29.5%)	—	—
Smoking status (%)						<0.001	0.372
Former	139 (14.4%)	441 (11.0%)	1492 (25.9%)	411 (41.4%)	495 (39.2%)	—	—
Never	771 (56.0%)	1352 (60.0%)	3512 (54.2%)	481 (47.2%)	525 (38.0%)	—	—
Now	199 (29.5%)	375 (29.0%)	1329 (19.9%)	139 (11.4%)	288 (22.8%)	—	—
Sleep duration (%)						<0.001	0.200
7–9 h	762 (71.7%)	1400 (69.5%)	3693 (61.5%)	655 (66.0%)	727 (59.8%)	—	—
<7 h	297 (24.0%)	690 (27.3%)	2347 (34.2%)	279 (24.9%)	479 (32.9%)	—	—
>9 h	50 (4.4%)	78 (3.2%)	293 (4.3%)	97 (9.1%)	102 (7.3%)	—	—
Sleep disorder (%)						<0.001	0.190
No	1082 (97.4%)	2096 (97.0%)	5953 (93.8%)	969 (94.4%)	1151 (87.1%)	—	—
Yes	27 (2.6%)	72 (3.0%)	380 (6.2%)	62 (5.6%)	157 (12.9%)	—	—
Trouble sleeping (%)						<0.001	0.260
No	926 (80.7%)	1768 (80.0%)	4635 (69.9%)	753 (70.3%)	737 (56.6%)	—	—
Yes	183 (19.3%)	400 (20.0%)	1698 (30.1%)	278 (29.7%)	571 (43.4%)	—	—
Hypertension (%)						<0.001	2.201
No	1109 (100.0%)	2168 (100.0%)	1928 (31.4%)	84 (6.7%)	205 (18.6%)	—	—
Yes	0.0 (0.0%)	0.0 (0.0%)	4405 (68.6%)	947 (93.3%)	1103 (81.4%)	—	—
Diabetes (%)						<0.001	0.762
No	1109 (100.0%)	2168 (100.0%)	5031 (83.8%)	504 (52.3%)	734 (61.3%)	—	—
Yes	0.0 (0.0%)	0.0 (0.0%)	1302 (16.2%)	527 (47.7%)	574 (38.7%)	—	—
CKD (%)						<0.001	0.621
No	1109 (100.0%)	2168 (100.0%)	5412 (87.3%)	632 (62.1%)	906 (73.2%)	—	—
Yes	0.0 (0.0%)	0.0 (0.0%)	921 (12.7%)	399 (37.9%)	402 (26.8%)	—	—
CVD (%)						<0.001	0.000
No	1109 (100.0%)	2168 (100.0%)	6333 (100.0%)	1031 (100.0%)	0.0 (0.0%)	—	—
Yes	0.0 (0.0%)	0.0 (0.0%)	0.0 (0.0%)	0.0 (0.0%)	1308 (100.0%)	—	—
SII quartile (%)						<0.001	0.271
Q1 (<311.17)	345 (29.7%)	625 (26.5%)	1530 (21.4%)	202 (16.2%)	285 (19.5%)	—	—
Q2 (311.17–434.75)	313 (30.3%)	574 (27.0%)	1610 (25.4%)	214 (20.3%)	275 (21.0%)	—	—
Q3 (434.75–619.88)	250 (22.6%)	548 (25.6%)	1594 (25.9%)	279 (29.0%)	318 (26.2%)	—	—
Q4 (>619.88)	201 (17.3%)	421 (20.9%)	1599 (27.2%)	336 (34.6%)	430 (33.3%)	—	—
SIRI quartile (%)						<0.001	0.482
Q1 (<0.64)	397 (33.2%)	663 (26.3%)	1566 (21.0%)	145 (11.1%)	180 (12.0%)	—	—
Q2 (0.64–0.94)	310 (30.4%)	592 (27.7%)	1664 (25.1%)	181 (16.9%)	239 (16.6%)	—	—
Q3 (0.94–1.40)	250 (23.4%)	513 (25.7%)	1635 (28.2%)	262 (27.0%)	318 (25.0%)	—	—
Q4 (>1.40)	152 (13.1%)	400 (20.3%)	1468 (25.6%)	443 (45.0%)	571 (46.4%)	—	—

*Note:* Median (IQR) for continuous variables: *p*‐value was calculated by the weighted linear regression model. Number (%) for categorical variables: *p*‐value was calculated by the weighted chi‐square test.

Abbreviations: BMI, body mass index; CKD, chronic kidney disease; CVD, cardiovascular disease; IQR, interquartile range; SII, systemic immune inflammation index; SIRI, systemic immune response index; SMD, standardized mean difference.

### 3.2. Association of Sleep With CKM Syndrome and Its Components

Table 2 displayed the associations between sleep pattern and CKM risk by the survey‐weighted logistic regression model. Through a tiered analysis involving three models, each incorporating increasing levels of adjustment for potential confounders, a consistent positive association was observed between poor sleep patterns and CKM syndrome, CKD, CVD, diabetes, and hypertension. Notably, after further adjusting for age, gender, race/ethnicity, education level, smoking status, drinking alcohol, and sedentary time, no significant correlation was demonstrated between sleep and metabolic syndrome (*p* > 0.05).

**Table 2:** Association of sleep pattern with CKM syndrome and its components.

Sleep pattern	Model 1		Model 2		Model 3	
	OR (95% CI)	*p*	OR (95% CI)	*p*	OR (95% CI)	*p*
Cardiovascular‐kidney‐metabolic syndrome						
Healthy	Ref	—	Ref	—	Ref	—
Intermediate	1.11 (0.97, 1.27)	0.145	1.05 (0.87, 1.27)	0.590	0.98 (0.80, 1.20)	0.834
Poor	2.11 (1.79, 2.49)	<0.001	2.42 (1.94, 3.01)	<0.001	1.88 (1.42, 2.47)	<0.001
Metabolic syndrome						
Healthy	Ref	—	Ref	—	Ref	—
Intermediate	1.29 (1.17, 1.43)	<0.001	1.30 (1.16, 1.45)	<0.001	1.35 (1.16, 1.57)	<0.001
Poor	1.96 (1.70, 2.26)	<0.001	1.96 (1.67, 2.29)	<0.001	1.26 (1.00, 1.58)	0.052
Chronic kidney disease						
Healthy	Ref	—	Ref	—	Ref	—
Intermediate	1.28 (1.10, 1.49)	0.002	1.18 (1.00, 1.40)	0.048	1.20 (1.00, 1.44)	0.056
Poor	1.67 (1.34, 2.07)	<0.001	1.42 (1.13, 1.80)	0.003	1.48 (1.12, 1.94)	0.006
Diabetes						
Healthy	Ref	—	Ref	—	Ref	—
Intermediate	1.25 (1.08, 1.45)	0.003	1.18 (1.01, 1.37)	0.034	1.08 (0.90, 1.29)	0.407
Poor	2.19 (1.79, 2.67)	<0.001	2.05 (1.66, 2.52)	<0.001	1.50 (1.16, 1.93)	0.002
Hypertension						
Healthy	Ref	—	Ref	—	Ref	—
Intermediate	1.36 (1.22, 1.52)	<0.001	1.28 (1.13, 1.45)	<0.001	1.33 (1.15, 1.54)	<0.001
Poor	2.55 (2.21, 2.95)	<0.001	2.30 (1.96, 2.70)	<0.001	2.00 (1.65, 2.44)	<0.001
Clinical Cardiovascular Disease						
Healthy	Ref	—	Ref	—	Ref	—
Intermediate	1.19 (0.98, 1.44)	0.083	1.13 (0.90, 1.41)	0.291	1.10 (0.87, 1.39)	0.412
Poor	2.81 (2.33, 3.39)	<0.001	2.74 (2.22, 3.38)	<0.001	2.21 (1.67, 2.92)	<0.001

*Note:* Model 1: no covariates were adjusted. Model 2: age, sex, race/ethnicity and education level were adjusted. Model 3: age, sex, race, education, BMI, waist circumference, smoking status, alcohol drinking status, and sedentary time were adjusted.

Abbreviations: CI, confidence intervals; OR, odds ratio.

To further validate the robustness of these findings and explore potential dose–response relationships, we conducted ordinal logistic regression, treating CKM Stages (0–4) as an ordered outcome (Table S8). After full adjustment for covariates, poor sleep patterns were significantly associated with more advanced CKM stages. Compared to a healthy sleep pattern, an intermediate sleep pattern was associated with 1.21‐fold higher odds of being in a more severe CKM stage (OR = 1.21, 95% CI: 1.08–1.35, *p*  < 0.001), and a poor sleep pattern was associated with 1.64‐fold higher odds (OR = 1.64, 95% CI: 1.37–1.95, *p*  < 0.001). The overall trend test for sleep patterns was highly significant (*p*  < 0.001), confirming a dose–response relationship across the full spectrum of CKM severity.

Furthermore, we employed RCS regression to investigate the relationship between sleep duration and CKM syndrome and its components (Figure 1). RCS regression analysis reveals nonlinear associations between sleep duration and CKM syndrome and its components. (*p* < 0.001).

Figure 1The RCS curve of the association between sleep hours and CKM syndrome and its components. (A) The RCS curve of the association between sleep hours and CKM. (B) The RCS curves of the association between sleep hours and metabolic syndrome. (C) The RCS curves of the association between sleep hours and CKD. (D) The RCS curves of the association between sleep hours and diabetes. (E) The RCS curves of the association between sleep hours and hypertension. (F) The RCS curves of the association between sleep hours and CVD. RCS regression was adjusted for age, sex, race, educational level, BMI, waist, smoking status, alcohol drinking, and sedentary time.

**Figure:**
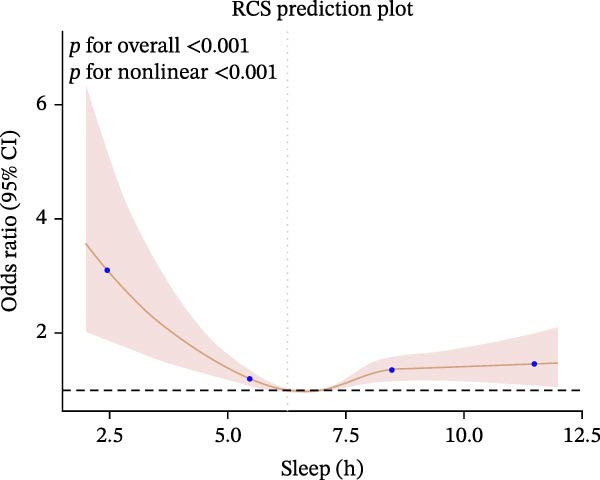
(A)

**Figure:**
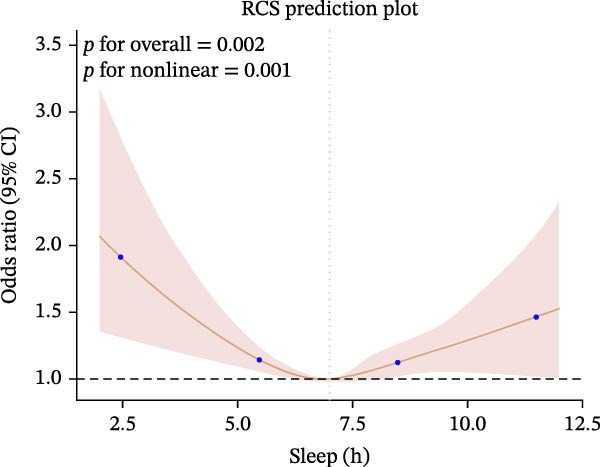
(B)

**Figure:**
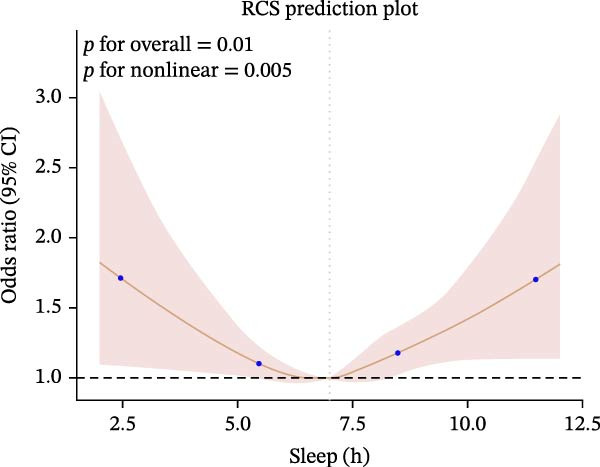
(C)

**Figure:**
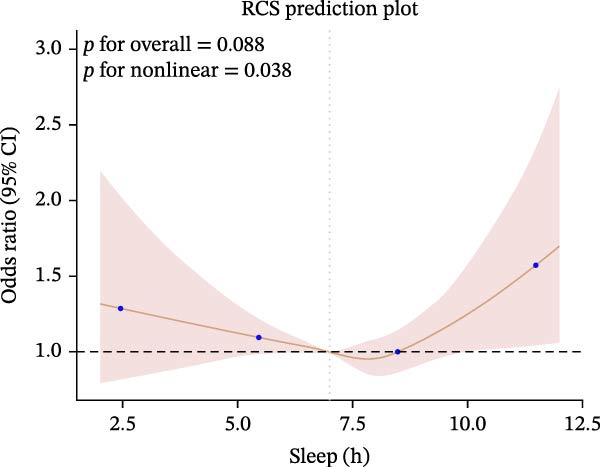
(D)

**Figure:**
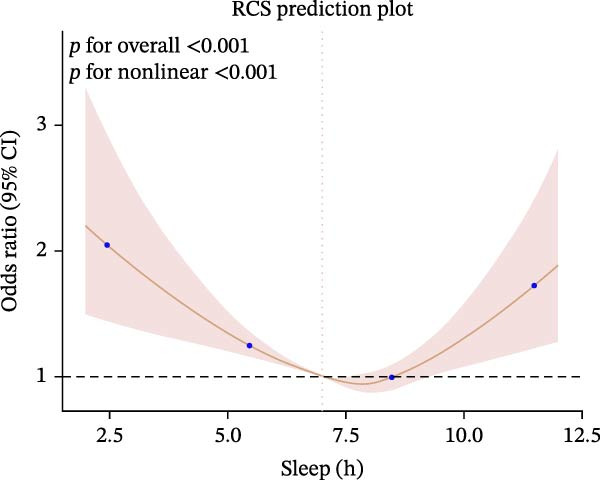
(E)

**Figure:**
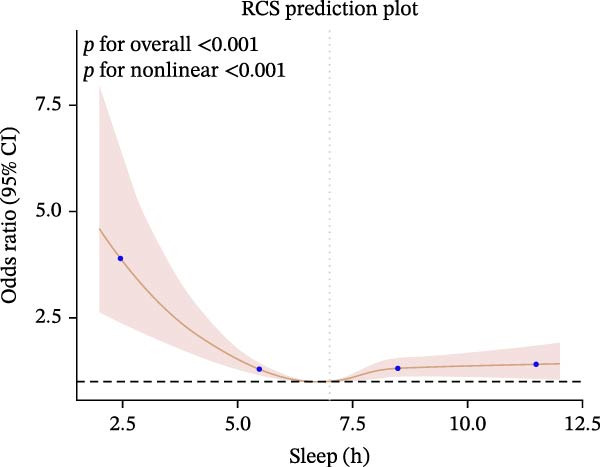
(F)

### 3.3. Associations Between Inflammatory Biomarkers and Sleep and CKM Syndrome

Table 3 showed the associations of inflammation biomarkers with sleep patterns based on the logistic regression. We found a significant correlation between SII and SIRI and sleep pattern after further adjusting for potential confounders (*p*  < 0.05). The results of multivariable logistic regression analysis between inflammation biomarkers and CKM syndrome displayed that SIRI was positively associated with CKM after adjusting for age, gender, race/ethnicity, education level, smoking status, drinking alcohol, and sedentary time (95%CI, 1.13, 1.41), while SII with no significant association (*p* = 0.290).

**Table 3:** The association between SII/SIRI and sleep and CKM syndrome.

Sleep pattern and CKM syndrome	Model 1		Model 2		Model 3	
	OR (95% CI)	*p*	OR (95% CI)	*p*	OR (95% CI)	*p*
Sleep pattern						
SII	1.00 (1.00, 1.00)	<0.001	1.00 (1.00, 1.00)	<0.001	1.00 (1.00, 1.00)	0.005
Quartile 1	Ref	—	Ref	—	Ref	—
Quartile 2	1.03 (0.90, 1.18)	0.622	0.99 (0.87, 1.14)	0.914	1.03 (0.89, 1.21)	0.672
Quartile 3	0.94 (0.81, 1.10)	0.461	0.93 (0.80, 1.08)	0.311	1.00 (0.84, 1.19)	0.998
Quartile 4	0.74 (0.64, 0.85)	<0.001	0.74 (0.64, 0.85)	<0.001	0.84 (0.71, 1.00)	0.053
P for trend	<0.001	—	<0.001	—	0.017	—
Per 1‐SD	0.84 (0.78, 0.91)	<0.001	0.85 (0.79, 0.92)	<0.001	0.89 (0.83, 0.97)	0.005
SIRI	0.87 (0.81, 0.93)	<0.001	0.87 (0.82, 0.94)	<0.001	0.93 (0.87, 1.00)	0.048
Quartile 1	Ref	—	Ref	—	Ref	—
Quartile 2	1.07 (0.95, 1.19)	0.261	1.01 (0.90, 1.12)	0.886	1.04 (0.91, 1.19)	0.567
Quartile 3	1.02 (0.91, 1.14)	0.733	0.97 (0.87, 1.08)	0.615	1.07 (0.93, 1.24)	0.338
Quartile 4	0.81 (0.70, 0.94)	0.006	0.80 (0.69, 0.93)	0.004	0.95 (0.80, 1.13)	0.577
P for trend	0.001	—	0.001	—	0.422	—
Per 1‐SD	0.88 (0.83, 0.94)	<0.001	0.89 (0.83, 0.94)	<0.001	0.94 (0.88, 1.00)	0..048
CKM syndrome						
SII	1.00 (1.00, 1.00)	<0.001	1.00 (1.00, 1.00)	0.050	1.00 (1.00, 1.00)	0.290
Quartile 1	Ref	—	Ref	—	Ref	—
Quartile 2	1.02 (0.84, 1.24)	0.827	1.15 (0.89, 1.47)	0.282	1.12 (0.83, 1.52)	0.435
Quartile 3	1.40 (1.17, 1.68)	<0.001	1.43 (1.17, 1.75)	<0.001	1.34 (1.04, 1.71)	0.021
Quartile 4	1.81 (1.48, 2.21)	<0.001	1.54 (1.22, 1.94)	<0.001	1.29 (0.96, 1.73)	0.094
P for trend	<0.001	—	<0.001	—	0.093	—
Per 1‐SD	1.33 (1.21, 1.46)	<0.001	1.05 (1.00, 1.33)	0.050	1.09 (0.93, 1.27)	0.290
SIRI	1.70 (1.57, 1.84)	<0.001	1.37 (1.25, 1.51)	<0.001	1.26 (1.13, 1.41)	<0.001
Quartile 1	Ref	—	Ref	—	Ref	—
Quartile 2	1.30 (1.03, 1.64)	0.027	1.30 (0.99, 1.70)	0.059	1.19 (0.86, 1.66)	0.284
Quartile 3	1.97 (1.59, 2.44)	<0.001	1.60 (1.24, 2.05)	<0.001	1.43 (1.09, 1.88)	0.011
Quartile 4	4.17 (3.34, 5.22)	<0.001	2.59 (2.00, 3.34)	<0.001	2.10 (1.54, 2.87)	<0.001
P for trend	<0.001	—	<0.001	—	<0.001	—
Per 1‐SD	1.60 (1.50, 1.72)	<0.001	1.33 (1.22, 1.44)	<0.001	1.23 (1.12, 1.36)	<0.001

*Note:* Model 1 was the crude model. Model 2 was adjusted for age, gender, race/ethnicity, and education level. Model 3 was further adjusted for BMI, waist circumference, smoking, drinking, and sedentary time.

### 3.4. Mediation Analysis

Figure 2 presents the direct and indirect effects of sleep disorder on CKM syndrome with SII and SIRI as mediators, respectively. Overall, both SII and SIRI mediated the relationship between self‐reported trouble sleeping and CKM syndrome, even after adjusting for confounders (*p* < 0.001, Table S9). Furthermore, the direct effects of the total sleep pattern and sleep disorder in relation to CKM syndrome were almost nonsignificant. As shown in Figure 2, SII explained 7.05%, 1.60%, and 0.73% of the association in Model 1, Model 2, and Model 3, respectively. Although the direct effects were all significant in these three cases (*p*  < 0.001), the indirect effects in Model 3 tended to be nonsignificant (*p* = 0.230). SIRI explained 17.59%, 10.55%, and 0.81% of the association in Model 1, Model 2, and Model 3, respectively (*p*  < 0.05). However, the mediating role of both SII and SIRI tended to be nonsignificant between sleep disorder and CKM syndrome in Model 3 (*p*  > 0.05). In addition, we conducted multiple mediation analysis to assess the mediating effects of SII and SIRI on the relationships between sleep patterns, sleep disorders, and CKM syndrome and its components (Tables S10–S13). After adjusting for factors in the primary analysis Model 3, the results showed that for trouble sleeping, the indirect effect proportions mediated by SIRI for CKM syndrome and CVD were 2.08% and 1.79%, respectively. For sleep patterns, the indirect effect proportions mediated by SII for CKM syndrome and CVD were −2.78% and −3.13%, respectively, while the indirect effect proportions mediated by SIRI were 8.33% and 3.13% (Figure S2).

Figure 2Estimated proportion of the association between self‐reported trouble sleeping and CKM syndrome mediated by SII (A–C), SIRI (D–F). Model 1 was the crude model (A, D); Model 2 was adjusted for age, gender, race/ethnicity, and education level (B, E); Model 3 was further adjusted for BMI, waist circumference, smoking, drinking, and sedentary time (C, F). IE, the estimate of the indirect effect; DE, the estimate of the direct effect; Proportion of mediation = IE/DE + IE.

**Figure:**
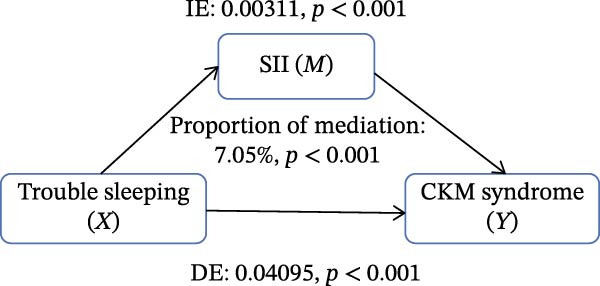
(A)

**Figure:**
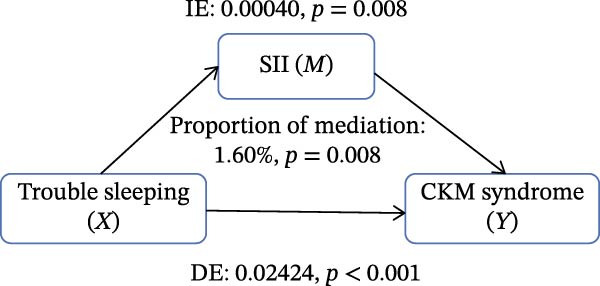
(B)

**Figure:**
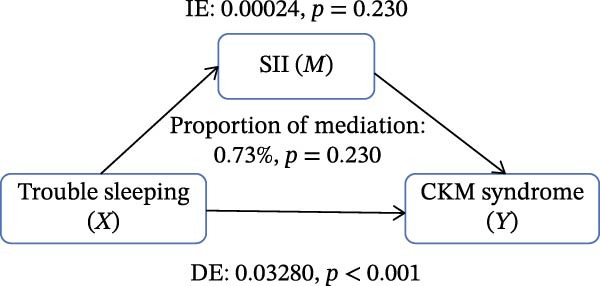
(C)

**Figure:**
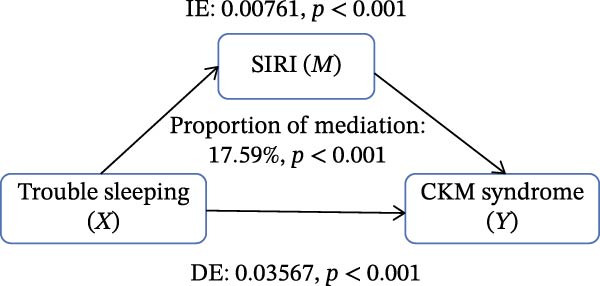
(D)

**Figure:**
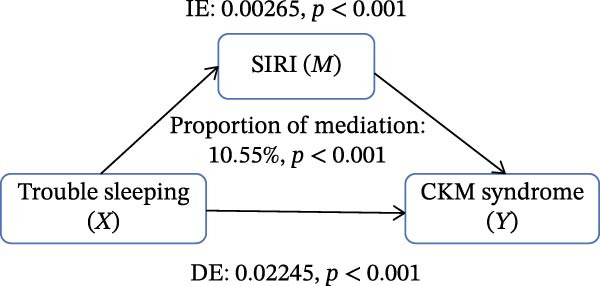
(E)

**Figure:**
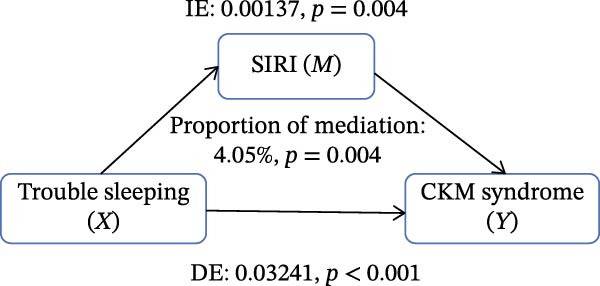
(F)

## 4. Discussion

This study is the first time to explore the significant relationship between sleep factors and CKM syndrome and its components using nationally representative data from the national sample. Our findings indicate that sleep factors, including sleep disorder, self‐reported trouble sleeping, and sleep duration, are significantly associated with CKM syndrome. We observed that a composite healthy sleep pattern was strongly linked to a lower likelihood of CKM, with evidence of a dose–response relationship. Exploratory mediation analyses suggested that systemic inflammatory indices (SII and SIRI) might partially underlie the association between subjective sleep complaints and CKM; however, this effect was substantially attenuated upon comprehensive adjustment for adiposity and lifestyle factors. Collectively, these findings underscore sleep health as a significant correlate of CKM risk and point to the complex interplay between sleep, metabolic dysregulation, and inflammation, warranting further investigation in longitudinal settings.

A significant association was observed in our research between poor sleep patterns with CKM and its components, while no significant relationship was observed with metabolic syndrome alone. Sleep has been linked to various health issues, impacting overall well‐being. Previous studies have extensively examined the relationship between sleep and related diseases. In adults, sleep disturbances and symptoms related to sleep, such as daytime sleepiness, nocturia, nocturnal dyspnea, and morning headaches, are common complaints [24]. These issues are often linked to CVD, poor adherence to medication, HF, and increased all‐cause mortality [25, 26]. The negative consequences of pathological sleep, whether characterized by poor quality or insufficient duration, are thoroughly documented, with short sleep duration recognized as a risk factor for cardiovascular and metabolic disorders [27]. Multiple epidemiological studies have indicated a link between short sleep duration and a heightened risk of developing type 2 diabetes mellitus (T2DM) and obesity [28, 29]. If the metabolic changes outlined persist, they can elevate the risk of obesity, hypercholesterolemia, metabolic syndrome, and diabetes, which in turn contributes to a higher likelihood of CVDs among short sleepers [30]. Several cohort studies have indicated that short sleep duration is linked to a rapid decline in kidney function and that unhealthy sleep patterns are associated with the progression of CKD [31, 32]. Apart from sleep duration, sleep factors, such as sleep disorder, is regarded as a risk factor for various conditions, including cardiovascular events [33, 34], hypertension [35], and type 2 diabetes [36]. In a large population‐based prospective cohort study, they found the healthy sleep score; no other sleep disorder was inversely associated with the risk of CKD in a dose‐dependent manner [37]. In our study, we found as sleep patterns worsened, there was a significant increase in the prevalence of CKM syndrome, CKD, metabolic syndrome, and CVDs, and the results were statistically significant.

It is well‐established that sleep plays a role in regulating the immune system, and there is evidence connecting sleep rhythm disruptions to heightened inflammation [38]. As indicators for assessing an individual’s systemic inflammatory activity, the higher the values of SII and SIRI, the more active the body’s immune system and inflammatory state. We selected the SII and SIRI as integrative biomarkers to probe this pathway. These indices move beyond single markers by combining differential white blood cell counts (and platelets for SII) to reflect the balance between pro‐inflammatory, thrombotic, and adaptive immune responses. A research study utilizing NHANES data investigated the connections among inflammatory markers, sleep disorders, and lifestyle habits. The results indicated a significant correlation between leukocyte inflammatory markers, including SII, and sleep disorders [39]. The mediation analysis yielded nuanced insights. While SII and SIRI showed significant indirect effects in initial models, a critical finding was the substantial attenuation or loss of significance of these effects after full adjustment for adiposity (BMI and waist circumference) and lifestyle factors. This attenuation is not merely a statistical adjustment but a pivotal biological insight. It suggests that the link between poor sleep and elevated systemic inflammation is largely embedded within shared upstream metabolic dysfunction, particularly central obesity. Adipose tissue is a potent endocrine organ that drives inflammation, obesity‐associated systemic inflammation may heighten vulnerability to mood disorders and disrupt circadian rhythms [40] and may be a primary risk factor for CKM components. Thus, our fully adjusted models indicate that what appeared as inflammatory mediation in simpler models may predominantly reflect the common causal effects of adiposity.

While traditional markers like CRP and IL‐6 have been widely used in previous research, SII/SIRI offer a more comprehensive view of systemic inflammation by integrating multiple hematological and inflammatory components. For instance, unlike CRP, which primarily reflects acute‐phase reactions, SII/SIRI incorporates neutrophil‐to‐lymphocyte ratio and PCs to capture chronic inflammatory states more holistically. Our results found that the quartile classifications of SII and SIRI both showed significant differences between healthy and poor sleep patterns. Additionally, the correlation analysis between the two measures and sleep patterns revealed statistically significant results, indicating a clear relationship between inflammatory indicators and sleep patterns. Significant relationships were observed between inflammatory markers and sleep disturbance in patients [41]. Notably, SIRI, which reflects myeloid cell activity, often demonstrated more robust associations than SII. This may offer a mechanistic clue: sleep disturbances are known to promote myelopoiesis and a pro‐inflammatory monocyte phenotype [38]. Given the central role of monocytes/macrophages in atherosclerosis, metabolic inflammation, and renal fibrosis [42], SIRI could be a more specific peripheral marker of sleep‐related activation of innate immunity relevant to CKM pathogenesis, whereas SII’s incorporation of platelets may reflect a broader state of thrombotic inflammation. Furthermore, a recent study highlights the significant associations between the SII and CKM syndrome and its various components, affirming the efficacy of SII as a key biomarker for assessing disease risk [43]. These findings prompted us to investigate the potential mediating role of inflammatory markers in the relationship between sleep and CKM syndrome. Notably, through mediation analysis, we discovered that both SII and SIRI significantly influenced this process in Models and Model 2, with no significant differences between sleep duration and sleep disorder and CKM syndrome after adjusting for Model 3. Therefore, while inflammation remains a plausible intermediary, our data reframe its role. Systemic inflammation, as captured by SII/SIRI, likely operates not as an independent mediator but as one component of a triadic network where sleep disturbance, metabolic dysregulation (especially obesity), and inflammatory activation are interconnected and mutually reinforcing. This network perspective necessarily implicates other parallel pathways, such as hormonal dysregulation and sympathetic nervous system activation, in linking sleep to CKM risk.

Based on the findings mentioned, we proceeded with further mediation analyses. We conducted mediation analysis separately for the components of sleep factors, including sleep patterns, sleep duration, sleep disorders, and self‐reported trouble sleeping in relation to CKM syndrome. When examining specific sleep components, a significant indirect effect through SII and SIRI was observed only for the relationship between self‐reported trouble sleeping and CKM syndrome in initial models (e.g., SII‐mediated proportion: 7.05%; SIRI: 17.59%). A pivotal finding, however, was the substantial attenuation of these effects upon full adjustment for adiposity and lifestyle factors in Model 3 (SII: 0.73%; SIRI: 4.05%), with the effect for SII becoming nonsignificant. However, there were no significant mediating effects on sleep patterns, sleep duration, and sleep disorders after adjusting for potential influencing factors. These findings indicate that although the inflammatory indicators, SII and SIRI, acted as mediators, adjusting the relationship between self‐reported trouble sleeping and CKM syndrome, they were highly sensitive to confounding by metabolic and behavioral covariates. The marked reduction in mediation magnitude after adjusting for factors like BMI and waist circumference suggests that obesity‐related metabolic dysfunction likely acts as a common upstream driver for both poor sleep (particularly subjective sleep dissatisfaction), elevated inflammatory tone, and CKM risk. Consequently, inflammation may not be an independent mediator but rather a downstream component of a shared pathway rooted in adiposity. The low residual mediation proportions further imply that other unmeasured or unadjusted pathways—such as neuroendocrine dysregulation, autonomic nervous system imbalance, or gut microbiota alterations—may contribute substantially to the sleep‐CKM link. Future mechanistic studies are warranted to dissect these complex, interrelated biological systems. Future research could explore these pathways through mechanistic studies to gain a more comprehensive understanding of the underlying mechanisms.

This study has several strengths. First, our research sample was drawn from a complex multistage probability sampling method, ensuring that the participants represent a large portion of the national population, which is suitable for the generalizability of the findings. Second, we explored the relationship between sleep patterns and CKM syndrome, demonstrating a significant association even after adjustments for multiple confounders. Last, based on previous research indicating, we explored a novel mechanistic pathway using integrative inflammatory indices (SII/SIRI) within a formal mediation framework. However, several important limitations must be emphasized, which contextualize the interpretation of our findings. First, the fundamental limitation is the cross‐sectional nature of the NHANES data, which precludes any causal inference regarding the directionality of the observed associations. While we conducted sensitivity analyses to mitigate concerns of reverse causation, the temporal sequence between sleep disturbances, inflammation, and CKM cannot be established. Prospective cohort or interventional studies are required to confirm causality. Second, all sleep measures were self‐reported and thus susceptible to recall and social desirability bias. The subjective measure of “trouble sleeping” may be particularly conflated with psychological distress (e.g., anxiety and depression), a potential unmeasured confounder. The lack of objective sleep measures is a notable weakness that prevents the assessment of objective sleep physiology and its relationship with CKM. Third, despite adjusting for a comprehensive set of covariates, the possibility of residual confounding persists. Factors such as detailed dietary patterns, psychosocial stress, genetic predisposition, and environmental exposures were not fully accounted for and could influence both sleep and CKM risk. Finally, the inflammatory indices SII and SIRI are systemic, nonspecific markers. Their levels are influenced by a wide array of conditions beyond sleep. The substantial attenuation of their mediating effect after adjusting for adiposity underscores that the observed association likely reflects a shared etiology with metabolic dysfunction, rather than a direct, independent inflammatory pathway. Our analysis cannot rule out other salient biological pathways (e.g., neuroendocrine dysregulation, and autonomic imbalance) that may link sleep to CKM.

## 5. Conclusion

In conclusion, this large, cross‐sectional study provides robust evidence that a healthy multidimensional sleep pattern is strongly associated with a lower risk and severity of CKM syndrome in a nationally representative adult population. Our exploration into potential mechanisms revealed a nuanced role for systemic inflammation. While integrative inflammatory indices (SII and SIRI) were correlated with both poor sleep and CKM, their apparent mediating effect was substantially attenuated and largely nonsignificant after rigorous adjustment for adiposity and lifestyle confounders. This key finding suggests that the relationship between sleep disturbances and inflammation is deeply intertwined with, and likely secondary to, shared underlying metabolic dysfunction. Therefore, our study supports a model in which sleep health is a significant, independent correlate of CKM risk, but its biological linkage may operate less through a direct inflammatory pathway than through a network of interconnected systems where metabolic health (particularly obesity) plays a central role. The specific mediation observed only for self‐reported trouble sleeping highlights the unique pathophysiology of subjective sleep dissatisfaction, which may be more closely tied to psychological and neuroendocrine stress axes that influence inflammation. Given these insights, future research should prioritize longitudinal studies to establish temporal and causal relationships. Mechanistic investigations are needed to disentangle the specific contributions of objective sleep deficiency versus subjective sleep perception and to clarify the roles of inflammation, neuroendocrine dysregulation, and autonomic function within the sleep‐CKM nexus. Ultimately, interventional trials are warranted to determine whether improving sleep can independently modify inflammatory profiles and, more importantly, slow the progression of CKM syndrome, thereby informing integrative strategies for CKM health.

## Author Contributions

**Minjie Hu**: writing – review and editing the manuscript. **Ying Xu and Jiao Ming**: prepare the figures. **Xuelin Zhang, Xiaolu Bian, Xinrui Liang, and Haiyan Wang**: review the manuscript. **Longyi Zheng, Ying Zhang, and Zhiyong Guo**: conceptualization, editing, visualization. All authors reviewed the manuscript.

## Funding

This work was supported by the Project of the Shanghai Municipal Science and Technology Commission (Grant 25Y12800104) to Zhiyong Guo, the Basic Medicine Project of Naval Medical University (Grant 2023MS016) to Longyi Zheng, the Basic Medicine Research Program of the First Affiliated Hospital of Naval Medical University (Grant 2023PY02) to Longyi Zheng, the Naval Medical University Nurse Funding (Grants 2022KYZ06, 2022KYG14, and 2022KYG20), and the Scientific research project of Shanghai Nursing Association (Grant 2024MS‐B08).

## Disclosure

All authors have seen and approved the manuscript being submitted, and contributed significantly to the work.

## Ethics Statement

The authors have nothing to report.

## Conflicts of Interest

The authors declare no conflicts of interest.

## Supporting Information

Additional supporting information can be found online in the Supporting Information section.

## Supporting information


**Supporting Information** Table S1: Weighted characteristics of the study population in disaggregated by sleep pattern. Table S2: Distribution of variables with missing data. Table S3: KDIGO risk for CKD classification. Table S4: Collinearity Statistics of covariates. Table S5: Baseline characteristics of participants within included and excluded group. Table S6: Subgroup analyses by excluding participants with CVD/CKD or medication use. Table S7: Negative control outcome analysis between SII and sleep pattern. Table S8: Associations between Sleep Patterns and Severity of CKM Syndrome: Results from Ordinal Logistic Regression. Table S9. The mediating effects of the relationship between trouble sleeping and CKM syndrome. Table S10: Systemic inflammation markers as mediators in the associations of sleep factors with CKM syndrome. Table S11: Systemic inflammation markers as mediators in the associations of sleep factors with Cardiovascular disease. Table S12: Systemic inflammation markers as mediators in the associations of sleep factors with Chronic kidney disease. Table S13: Systemic inflammation markers as mediators in the associations of sleep factors with Metabolic syndrome. Figure S1: Flowchart showing the selection of the studied population. Figure S2: Mediation analysis of sleep pattern, sleep disorder and trouble sleeping with CKM syndrome, CVD, CKD, and Mets.

## Data Availability

The data that support the findings of this study are available in NHANES at https://www.cdc.gov/nchs/nhanes. These data were derived from the following resources available in the public domain https://www.cdc.gov/nchs/nhanes.
