# Supplementary material for: Association Between Sleep and Cardiovascular‐Kidney‐Metabolic Syndrome: Mediation Analysis of Inflammatory Biomarkers
Source: Mediators Inflamm. 2026 Apr 24;2026:9072641. doi: 10.1155/mi/9072641 (PMC13108432; doi:10.1155/mi/9072641)
Supplement: Supplementary file 1 — Supporting Information Table S1: Weighted characteristics of the study population in disaggregated by sleep pattern. Table S2: Distribution of variables with missing data. Table S3: KDIGO risk for CKD classification. Table S4: Collinearity Statistics of covariates. Table S5: Baseline characteristics of participants within included and excluded group. Table S6: Subgroup analyses by excluding participants with CVD/CKD or medication use. Table S7: Negative control outcome analysis between SII and sleep pattern. Table S8: Associations between Sleep Patterns and Severity of CKM Syndrome: Results from Ordinal Logistic Regression. Table S9. The mediating effects of the relationship between trouble sleeping and CKM syndrome. Table S10: Systemic inflammation markers as mediators in the associations of sleep factors with CKM syndrome. Table S11: Systemic inflammation markers as mediators in the associations of sleep factors with Cardiovascular disease. Table S12: Systemic inflammation markers as mediators in the associations of sleep factors with Chronic kidney disease. Table S13: Systemic inflammation markers as mediators in the associations of sleep factors with Metabolic syndrome. Figure S1: Flowchart showing the selection of the studied population. Figure S2: Mediation analysis of sleep pattern, sleep disorder and trouble sleeping with CKM syndrome, CVD, CKD, and Mets. [file MI-2026-9072641-s001.docx]

**Supplemental materials**

**Association between sleep and cardiovascular-kidney-metabolic syndrome: mediation analysis of inflammatory biomarkers**

Minjie Hu^a^^#^, Ying Xu^b#^, Jiao Ming^a#^, Xuelin Zhang^a^, Xiaolu Bian^a^, Xinrui Liang^a^, Haiyan Wang^a^, Longyi Zheng^c^, Ying Zhang^d^, Zhiyong Guo^a^

**Table S1** Weighted characteristics of the study population in disaggregated by sleep pattern. (n=11,949).

**Table S2** Distribution of variables with missing data. (n=22,461).

**Table S3** KDIGO risk for CKD classification.

**Table S4** Collinearity Statistics of covariates.

**Table S5** Baseline characteristics of participants within included and excluded ( Age ≥20) group.

**Table S6** Subgroup analyses by excluding participants with CVD/CKD or medication use.

**Table S7** Negative control outcome analysis between SII and sleep pattern.

**Table S8** Associations between Sleep Patterns and Severity of CKM Syndrome: Results from Ordinal Logistic Regression.

**Table S9** The mediating effects of the relationship between trouble sleeping and CKM syndrome.

**Table S10** Systemic inflammation markers as mediators in the associations of sleep factors with CKM syndrome.

**Table S11** Systemic inflammation markers as mediators in the associations of sleep factors with Cardiovascular disease.

**Table S12** Systemic inflammation markers as mediators in the associations of sleep factors with Chronic kidney disease.

**Table S13** Systemic inflammation markers as mediators in the associations of sleep factors with Metabolic syndrome.

**Figure S1** Flowchart showing the selection of the studied population.

**Figure S2** Mediation analysis of sleep pattern, sleep disorder and trouble sleeping with CKM syndrome, CVD, CKD, and Mets.

**Table S1 Weighted characteristics of the study population in disaggregated by sleep pattern (n=11,949).**

|  | Healthy | Intermediate | Poor | P value |
| --- | --- | --- | --- | --- |
| N | 5632 | 4434 | 1883 |  |
| Age (median [IQR]) | 45.00 [31.00, 60.00] | 47.00 [35.00, 60.00] | 51.62 [39.05, 61.00] | <0.001 |
| **Gender (%)** |  |  |  |  |
| male | 2958 (52.4) | 2223 (50.0) | 849 (46.4) | 0.003 |
| female | 2674 (47.6) | 2211 (50.0) | 1034 (53.6) |  |
| **Race (%)** |  |  |  |  |
| Mexican American | 975 (9.6) | 655 (8.2) | 208 (5.8) | <0.001 |
| Other Hispanic | 644 (6.0) | 487 (6.0) | 198 (4.8) |  |
| Non-Hispanic White | 2371 (67.9) | 1737 (63.7) | 909 (71.5) |  |
| Non-Hispanic Black | 870 (8.4) | 994 (12.9) | 432 (13.1) |  |
| Other Race | 722 (8.0) | 561 (9.2) | 136 (4.7) |  |
| **Education (%)** |  |  |  |  |
| less than high school | 1349 (15.3) | 1091 (16.4) | 486 (17.5) | 0.001 |
| high school | 1242 (21.0) | 1016 (24.2) | 441 (25.0) |  |
| more than high school | 3041 (63.7) | 2327 (59.4) | 956 (57.5) |  |
| BMI (median [IQR]) | 27.40 [23.80, 31.60] | 27.80 [24.17, 32.20] | 30.10 [25.56, 35.47] | <0.001 |
| Waist (median [IQR]) | 96.50 [86.40, 107.30] | 97.40 [87.10, 108.30] | 103.82 [92.50, 117.30] | <0.001 |
| **CKM syndrome (%)** |  |  |  |  |
| stage0 | 657 (14.0) | 361 (10.2) | 91 (6.2) | <0.001 |
| stage1 | 1169 (24.5) | 794 (20.3) | 205 (11.9) |  |
| stage2 | 2844 (49.4) | 2450 (56.1) | 1039 (59.2) |  |
| stage3 | 495 (5.4) | 378 (5.3) | 158 (5.7) |  |
| stage4 | 467 (6.8) | 451 (8.0) | 390 (17.0) |  |
| **Sleep duration (%)** |  |  |  |  |
| 7-9hours | 5632 (100.0) | 1385 (38.0) | 220 (13.7) | <0.001 |
| <7hours | 0.0 (0.0) | 2613 (53.2) | 1479 (77.0) |  |
| >9hours | 0.0 (0.0) | 436 (8.7) | 184 (9.3) |  |
| **Sleep disorder (%)** |  |  |  |  |
| no | 5632 (100.0) | 4360 (98.1) | 1259 (67.2) | <0.001 |
| yes | 0.0 (0.0) | 74 (1.9) | 624 (32.8) |  |
| **Trouble sleeping (%)** |  |  |  |  |
| no | 5632 (100.0) | 3123 (63.8) | 64 (2.9) | <0.001 |
| yes | 0.0 (0.0) | 1311 (36.2) | 1819 (97.1) |  |
| **Smoking status(%)** |  |  |  |  |
| former | 1379 (25.2) | 1096 (25.6) | 503 (28.1) | <0.001 |
| never | 3373 (60.0) | 2397 (53.3) | 871 (43.9) |  |
| now | 880 (14.7) | 941 (21.1) | 509 (28.0) |  |
| **Low physical activity (%)** |  |  |  |  |
| no | 190 (4.2) | 168 (4.4) | 46 (2.6) | 0.034 |
| yes | 5442 (95.8) | 4266 (95.6) | 1837 (97.4) |  |
| **Obesity (%)** |  |  |  |  |
| no | 1842 (33.5) | 1262 (30.1) | 396 (22.1) | <0.001 |
| yes | 3790 (66.5) | 3172 (69.9) | 1487 (77.9) |  |
| **Abdominal obesity (%)** |  |  |  |  |
| no | 2448 (44.1) | 1798 (41.4) | 617 (33.0) | <0.001 |
| yes | 3184 (55.9) | 2636 (58.6) | 1266 (67.0) |  |
| **Hypertension (%)** |  |  |  |  |
| no | 2913 (57.4) | 1983 (49.8) | 598 (34.6) | <0.001 |
| yes | 2719 (42.6) | 2451 (50.2) | 1285 (65.4) |  |
| **Diabetes (%)** |  |  |  |  |
| no | 4663 (88.0) | 3983 (85.4) | 1363 (77.0) | <0.001 |
| yes | 969 (12.0) | 451 (14.6) | 520 (23.0) |  |
| **CKD (%)** |  |  |  |  |
| no | 4903 (90.5) | 3772 (88.1) | 1552 (85.1) | <0.001 |
| yes | 729 (9.5) | 662 (11.9) | 331 (14.9) |  |
| **CVD (%)** |  |  |  |  |
| no | 5165 (93.2) | 3983 (92.0) | 1493 (83.0) | <0.001 |
| yes | 467 (6.8) | 451 (8.0) | 390 (17.0) |  |
| **SII quartile (%)** |  |  |  |  |
| Q1 | 1458 (24.0) | 1119 (23.7) | 410 (18.0) | <0.001 |
| Q2 | 1463 (27.2) | 1066 (24.1) | 457 (24.3) |  |
| Q3 | 1421 (26.0) | 1083 (24.5) | 485 (27.4) |  |
| Q4 | 1290 (22.8) | 1166 (27.7) | 531 (30.2) |  |
| **SIRI quartile (%)** |  |  |  |  |
| Q1 | 1407 (22.6) | 1131 (23.5) | 413 (17.9) | <0.001 |
| Q2 | 1450 (26.3) | 1075 (24.2) | 461 (23.2) |  |
| Q3 | 1471 (27.5) | 1061 (26.0) | 446 (26.3) |  |
| Q4 | 1304 (23.6) | 1167 (26.2) | 563 (32.7) |  |

Median (IQR) for continuous variables: P-value was calculated by the weighted linear regression model. Number(%) for categorical variables: P-value was calculated by the weighted chi-square test.

IQR, interquartile range; BMI, body mass index; CKD, chronic kidney disease; CVD, cardiovascular disease; SII, systemic immune inflammation index; SIRI, systemic immune response index.

**Table S2** **Distribution of variables with missing data. (n=22,461)**

| variables | Number of Missing | Missing proportion |
| --- | --- | --- |
| Sample weights (WTSAF2YR) | 20286 | 33.90% |
| BMI | 165 | 0.28% |
| Waist circumstance | 395 | 0.66% |
| HDL-c | 126 | 0.21% |
| Serum creatinine | 57 | 0.10% |
| Smoking status | 29 | 0.05% |
| Blood pressure | 90 | 0.15% |
| SII/SIRI | 1393 | 2.33% |

Abbreviations: BMI, body mass index; SII, systemic immune inflammation index; SIRI, systemic immune response index.

**Table S3 KDIGO risk for CKD classification.**

| eGFR categories  ml/min per 1.73 m^2^ | | | Albuminuria categories | | |
| --- | --- | --- | --- | --- | --- |
|  |  |  | A1 | A2 | A3 |
|  |  |  | Normal to mildly increased | Moderately increased | Severely increased |
|  |  |  | <30 mg/g | 30-299 mg/g | ≥300 mg/g |
| G1 | Normal or high | ≥90 | Low risk | Moderately increased risk | High risk |
| G2 | Mildly decreases | 60-89 | Low risk | Moderately increased risk | High risk |
| G3a | Mildly to moderately decreased | 45-59 | Moderately increased risk | High risk | Very high risk |
| G3b | Moderately to severely decrease | 30-44 | High risk | Very high risk | Very high risk |
| G4 | Severely decreased | 15-29 | Very high risk | Very high risk | Very high risk |
| G5 | Kidney failure | <15 | Very high risk | Very high risk | Very high risk |

Abbreviations: CKD, chronic kidney disease; eGFR, estimated glomerular filtration rate; KDIGO, Kidney Disease Improving Global Outcomes.

**Table S4 Collinearity Statistics.**

| Variables | GVIF | Df | GVIF ^1/2Df^ |
| --- | --- | --- | --- |
| Sleep pattern | 1.704406 | 1 | 1.305529 |
| Sleep hours | 1.157457 | 1 | 1.075852 |
| Sleep disorder | 1.124560 | 1 | 1.060453 |
| Trouble sleeping | 1.634777 | 1 | 1.278584 |
| Age | 1.390337 | 1 | 1.179126 |
| Sex | 1.416034 | 1 | 1.189972 |
| Race | 1.432869 | 4 | 1.045986 |
| Education level | 1.258956 | 2 | 1.059260 |
| Smoking status | 1.297474 | 2 | 1.067271 |
| Alcohol drinking | 1.275367 | 3 | 1.041372 |
| BMI | 8.132274 | 1 | 2.851714 |
| Waist circumstance | 8.563368 | 1 | 2.926323 |
| SII | 2.316734 | 1 | 1.522082 |
| SIRI | 2.427468 | 1 | 1.558033 |
| Sedentary time | 1.070554 | 1 | 1.034676 |

Abbreviations: BMI, body mass index; SII, systemic immune inflammation index; SIRI, systemic immune response index.

**Table S9 The mediating effects of the relationship between trouble sleeping and CKM syndrome.**

|  | SII  Estimation  (95%CI) | P-value | SIRI  Estimation  (95%CI) | P-value |
| --- | --- | --- | --- | --- |
| Model 1 | | | | |
| TE | 0.074 (0.06,0.09) | <0.001 | 0.072 (0.06,0.09) | <0.001 |
| DE | 0.071 (0.05,0.09) | <0.001 | 0.063 (0.05,0.08) | <0.001 |
| IE | 0.003 (0.00,0.01) | <0.001 | 0.008 (0.00,0.01) | <0.001 |
| Mediation proportion, % | 7.05 (0.04,0.09) | <0.001 | 17.60 (0.09,0.19) | <0.001 |
| Model 2 | | | | |
| TE | 0.045 (0.03,0.06) | <0.001 | 0.045 (0.03,0.06) | <0.001 |
| DE | 0.040 (0.03,0.05) | <0.001 | 0.039 (0.03,0.05) | <0.001 |
| IE | 0.000 (0.00,0.00) | 0.008 | 0.001 (0.00,0.01) | <0.001 |
| Mediation proportion, % | 1.60 (0.01,0.05) | 0.008 | 10.55 (0.04,0.10) | <0.001 |
| Model 3 | | | | |
| TE | 0.033 (0.02,0.04) | <0.001 | 0.034 (0.01,0.04) | <0.001 |
| DE | 0.033 (0.02,0.04) | <0.001 | 0.032 (0.02,0.04) | 0.034 |
| IE | 0.000 (-0.00,0.00) | 0.230 | 0.001 (0.00,0.01) | 0.004 |
| Mediation proportion, % | 0.73 (-0.01,0.03) | 0.230 | 4.05 (0.01,0.08) | 0.004 |

Abbreviations: SII, systemic immune inflammation index; SIRI, systemic immune response index

**Table S10 Systemic inflammation markers as mediators in the associations of sleep factors with CKM syndrome.**

|  | Indirect effects  β (Boot 95%CI) | Direct effects  β (Boot 95%CI) | Mediated proportion (%) | P-value |
| --- | --- | --- | --- | --- |
| Sleep pattern | | | | |
| Model 1 |  |  |  |  |
| SII | 0.001 (0.002,0.005) | -0.039 (-0.053,-0.025) | 6.70 | <0.001 |
| SIRI | -0.009 (-0.013, -0.005) | -0.041(-0.055, -0.026) | 20.00 | <0.001 |
| Model 2 |  |  |  |  |
| SII | 0.001 (-0.000,0.001) | -0.034 (-0.043,-0.023) | 2.78 | 0.025 |
| SIRI | -0.003 (-0.004,-0.002) | -0.035 (-0.045,-0.024) | 8.33 | <0.001 |
| Model 3 |  |  |  |  |
| SII | 0.000 (-0.000,0.001) | -0.021 (-0.033,-0.010) | 0.00 | 0.265 |
| SIRI | -0.001 (-0.002,-0.000) | -0.022 (-0.033,-0.011) | 4.35 | 0.025 |
| Sleep disorder | | | | |
| Model 1 |  |  |  |  |
| SII | -0.004 (-0.008,-0.001) | 0.103 (0.069,0.137) | 3.33 | 0.025 |
| SIRI | 0.021 (0.010,0.033) | 0.110 (0.075,0.143) | 17.50 | <0.001 |
| Model 2 |  |  |  |  |
| SII | -0.001 (-0.002,0.000) | 0.081 (0.056,0.103) | 1.19 | 0.159 |
| SIRI | 0.004 (0.001,0.008) | 0.082 (0.057,0.105) | 4.76 | 0.024 |
| Model 3 |  |  |  |  |
| SII | 0.000 (-0.000,0.001) | 0.045 (0.021, 0.072) | 0.00 | 0.683 |
| SIRI | -0.000 (-0.003,0.003) | 0.046 (0.022,0.073) | 0.00 | 0.949 |
| Trouble sleeping | | | | |
| Model 1 |  |  |  |  |
| SII | -0.006 (-0.009,-0.003) | 0.087 (0.071,0.104) | 6.19 | <0.001 |
| SIRI | 0.016 (0.011,0.022) | 0.092 (0.075,0.110) | 16.49 | <0.001 |
| Model 2 |  |  |  |  |
| SII | -0.001 (-0.001,0.000) | 0.058 (0.045,0.070) | 1.64 | 0.050 |
| SIRI | 0.004 (0.002,0.006) | 0.059 (0.046,0.072) | 6.56 | <0.001 |
| Model 3 |  |  |  |  |
| SII | -0.000 (-0.001,0.000) | 0.047 (0.034,0.060) | 0.00 | 0.504 |
| SIRI | 0.001 (0.000,0.003) | 0.048 (0.034,0.060) | 2.08 | 0.034 |

Abbreviations: SII, systemic immune inflammation index; SIRI, systemic immune response index.

**Table S11 Systemic inflammation markers as mediators in the associations of sleep factors with Cardiovascular disease.**

|  | Indirect effects  β (Boot 95%CI) | Direct effects  β (Boot 95%CI) | Mediated proportion (%) | P-value |
| --- | --- | --- | --- | --- |
| Sleep pattern | | | | |
| Model 1 |  |  |  |  |
| SII | 0.002 (0.001,0.004) | -0.047 (-0.058,-0.036) | 4.00 | 0.002 |
| SIRI | -0.005 (-0.007, -0.003) | -0.049(-0.060, -0.037) | 10.64 | <0.001 |
| Model 2 |  |  |  |  |
| SII | 0.001 (0.000,0.002) | -0.042 (-0.051,-0.032) | 2.33 | 0.026 |
| SIRI | -0.002 (-0.004,-0.001) | -0.042 (-0.053,-0.032) | 4.65 | <0.001 |
| Model 3 |  |  |  |  |
| SII | 0.001 (0.000,0.002) | -0.032 (-0.042,-0.021) | 3.13 | 0.030 |
| SIRI | -0.001 (-0.002,-0.000) | -0.032 (-0.043,-0.021) | 3.13 | 0.033 |
| Sleep disorder | | | | |
| Model 1 |  |  |  |  |
| SII | -0.003 (-0.006,-0.001) | 0.108 (0.077,0.138) | 2.54 | 0.036 |
| SIRI | 0.013 (0.006,0.021) | 0.116 (0.083,0.147) | 12.04 | <0.001 |
| Model 2 |  |  |  |  |
| SII | -0.001 (-0.002,0.000) | 0.090 (0.063,0.115) | 1.09 | 0.199 |
| SIRI | 0.003 (0.001,0.007) | 0.092 (0.065,0.118) | 3.26 | 0.032 |
| Model 3 |  |  |  |  |
| SII | 0.000 (-0.001,0.002) | 0.036 (0.012, 0.064) | 0.00 | 0.595 |
| SIRI | -0.000 (-0.002,0.002) | 0.037 (0.012,0.065) | 0.00 | 0.949 |
| Trouble sleeping | | | | |
| Model 1 |  |  |  |  |
| SII | -0.004 (-0.006,-0.002) | 0.092 (0.077,0.106) | 4.12 | <0.001 |
| SIRI | 0.009 (0.006,0.013) | 0.097 (0.082,0.112) | 9.28 | <0.001 |
| Model 2 |  |  |  |  |
| SII | -0.001 (-0.002,-0.000) | 0.073 (0.061,0.086) | 1.33 | 0.044 |
| SIRI | 0.003 (0.002,0.005) | 0.075 (0.062,0.088) | 4.00 | <0.001 |
| Model 3 |  |  |  |  |
| SII | -0.001 (-0.001,0.000) | 0.056 (0.043,0.070) | 1.79 | 0.223 |
| SIRI | 0.001 (0.000,0.003) | 0.057 (0.043,0.071) | 1.79 | 0.040 |

Abbreviations: SII, systemic immune inflammation index; SIRI, systemic immune response

**Table S12 Systemic inflammation markers as mediators in the associations of sleep factors with Chronic kidney disease.**

|  | Indirect effects  β (Boot 95%CI) | Direct effects  β (Boot 95%CI) | Mediated proportion (%) | P-value |
| --- | --- | --- | --- | --- |
| Sleep pattern | | | | |
| Model 1 |  |  |  |  |
| SII | -0.000 (-0.001,0.000) | -0.024 (-0.036,-0.011) | 0.00 | 0.428 |
| SIRI | -0.002 (-0.004, -0.001) | -0.024 (-0.036,-0.011) | 7.69 | <0.001 |
| Model 2 |  |  |  |  |
| SII | -0.001 (-0.002,0.000) | -0.014 (-0.026,-0.002) | 6.25 | 0.100 |
| SIRI | -0.001 (-0.002,-0.001) | -0.014 (-0.026,-0.002) | 6.25 | 0.004 |
| Model 3 |  |  |  |  |
| SII | -0.000 (-0.001,0.000) | -0.011 (-0.024,-0.002) | 0.00 | 0.228 |
| SIRI | -0.001 (-0.001,-0.000) | -0.011 (-0.024,-0.002) | 8.33 | 0.067 |
| Sleep disorder | | | | |
| Model 1 |  |  |  |  |
| SII | 0.000 (-0.000,0.002) | 0.031 (0.002,0.059) | 0.00 | 0.411 |
| SIRI | 0.006 (0.002,0.009) | 0.031 (0.002,0.060) | 16.22 | 0.001 |
| Model 2 |  |  |  |  |
| SII | 0.001 (-0.000,0.002) | 0.023 (-0.005,0.050) | 2.70 | 0.199 |
| SIRI | 0.002 (0.000,0.004) | 0.023 (-0.005,0.050) | 5.41 | 0.053 |
| Model 3 |  |  |  |  |
| SII | -0.000 (-0.001,0.001) | 0.012 (-0.015, 0.041) | 0.00 | 0.643 |
| SIRI | -0.000 (-0.002,0.002) | 0.012 (-0.015,0.042) | 0.00 | 0.952 |
| Trouble sleeping | | | | |
| Model 1 |  |  |  |  |
| SII | 0.001 (-0.001,0.002) | 0.025 (0.009,0.040) | 3.33 | 0.391 |
| SIRI | 0.004 (0.003,0.006) | 0.025 (0.009,0.040) | 13.33 | <0.001 |
| Model 2 |  |  |  |  |
| SII | 0.001 (-0.000,0.002) | 0.007 (-0.008,0.020) | 10.00 | 0.093 |
| SIRI | 0.002 (0.001,0.003) | 0.007 (-0.008,0.020) | 20.00 | 0.005 |
| Model 3 |  |  |  |  |
| SII | 0.000 (-0.000,0.001) | 0.006 (-0.008,0.021) | 0.00 | 0.358 |
| SIRI | 0.001 (0.000,0.002) | 0.006 (-0.008,0.021) | 14.29 | 0.075 |

Abbreviations: SII, systemic immune inflammation index; SIRI, systemic immune response index.

**Table S13 Systemic inflammation markers as mediators in the associations of sleep factors with Metabolic syndrome.**

|  | Indirect effects  β (Boot 95%CI) | Direct effects  β (Boot 95%CI) | Mediated proportion (%) | P-value |
| --- | --- | --- | --- | --- |
| Sleep pattern | | | | |
| Model 1 |  |  |  |  |
| SII | 0.002 (0.001,0.004) | -0.069 (-0.086,-0.050) | 2.70 | 0.028 |
| SIRI | -0.007 (-0.011, -0.004) | -0.069 (-0.086,-0.050) | 9.46 | <0.001 |
| Model 2 |  |  |  |  |
| SII | -0.002 (-0.003,-0.000) | -0.066 (-0.082,-0.048) | 2.86 | 0.022 |
| SIRI | -0.002 (-0.003,-0.000) | -0.066 (-0.082,-0.048) | 2.86 | 0.044 |
| Model 3 |  |  |  |  |
| SII | -0.000 (-0.001,0.001) | -0.026 (-0.040,-0.010) | 0.00 | 0.621 |
| SIRI | -0.000 (-0.001,0.000) | -0.026 (-0.040,-0.010) | 0.00 | 0.620 |
| Sleep disorder | | | | |
| Model 1 |  |  |  |  |
| SII | -0.002 (-0.005,-0.000) | 0.129 (0.092,0.165) | 1.41 | 0.084 |
| SIRI | 0.015 (0.007,0.024) | 0.128 (0.091,0.164) | 10.56 | <0.001 |
| Model 2 |  |  |  |  |
| SII | 0.001 (-0.000,0.004) | 0.104 (0.068,0.139) | 0.93 | 0.153 |
| SIRI | 0.002 (0.000,0.005) | 0.104 (0.068,0.139) | 1.87 | 0.116 |
| Model 3 |  |  |  |  |
| SII | -0.000 (-0.001,0.001) | -0.035 (-0.071, 0.002) | 0.00 | 0.804 |
| SIRI | -0.000 (-0.001,0.001) | -0.035 (-0.071, 0.002) | 0.00 | 0.974 |
| Trouble sleeping | | | | |
| Model 1 |  |  |  |  |
| SII | -0.003 (-0.006,-0.001) | 0.094 (0.074,0.113) | 2.91 | 0.010 |
| SIRI | 0.012 (0.008,0.018) | 0.093 (0.073,0.112) | 11.65 | <0.001 |
| Model 2 |  |  |  |  |
| SII | 0.002 (0.000,0.004) | 0.094 (0.074,0.112) | 2.04 | 0.024 |
| SIRI | 0.002 (0.000,0.004) | 0.094 (0.074,0.112) | 2.04 | 0.053 |
| Model 3 |  |  |  |  |
| SII | 0.000 (-0.000,0.001) | 0.031 (0.012,0.051) | 0.00 | 0.660 |
| SIRI | 0.000 (-0.001,0.001) | 0.031 (0.012,0.051) | 0.00 | 0.659 |

Abbreviations: SII, systemic immune inflammation index; SIRI, systemic immune response index.

**Table S6 Subgroup analyses by excluding participants with CVD/CKD or medication use**

|  | Model 1 | | Model 2 | | Model 3 | |
| --- | --- | --- | --- | --- | --- | --- |
|  | OR (95%CI) | P | OR (95%CI) | P | OR (95%CI) | P |
| No CVD (N=10641) | | | | | | |
| healthy | ref |  | ref |  | ref |  |
| intermediate | 1.00(0.84,1.20) | 0.965 | 1.00(0.80,1.25) | 0.980 | 0.82(0.62,1.08) | 0.149 |
| poor | 1.19(0.90,1.58) | 0.210 | 1.56(1.07,2.30) | 0.023 | 1.39(0.94,2.05） | 0.093 |
| No CKD (N=10227) | | | | | | |
| healthy | ref |  | ref |  | ref |  |
| intermediate | 1.10(0.92,1.31) | 0.312 | 1.02(0.82,1.27) | 0.875 | 0.98(0.78,1.23) | 0.852 |
| poor | 2.08(1.73,2.49) | <0.001 | 2.13(1.68,2.70) | <0.001 | 1.69(1.26,2.27） | 0.001 |
| No β-Blocker use (N=11942) | | | | | | |
| healthy | ref |  | ref |  | ref |  |
| intermediate | 1.10(0.96,1.26) | 1.154 | 1.05(0.87,1.27) | 0.610 | 0.98(0.80,1.20) | 0.826 |
| poor | 2.11(1.79,2.49) | <0.001 | 2.41(1.94,3.01) | <0.001 | 1.89(1.43,2.49) | <0.001 |
| No steroid use (N=11926) | | | | | | |
| healthy | ref |  | ref |  | ref |  |
| intermediate | 1.10(0.96,1.26) | 0.168 | 1.04(0.86,1.26) | 0.647 | 0.97(0.80,1.19) | 0.803 |
| poor | 2.10(1.78,2.47) | <0.001 | 2.37(1.91,2.94) | <0.001 | 1.84(1.40,2.41) | <0.001 |

OR, odds ratio; CI, confidence intervals.

Model 1: no covariates were adjusted.

Model 2: age, sex, race/ethnicity and education level were adjusted.

Model 3: age, sex, race, education, BMI, waist circumference, smoking status, alcohol drinking status and sedentary time were adjusted.

**Table S7** **Negative control outcome analysis between SII and sleep pattern**

|  | Model 1 | | Model 2 | | Model 3 | |
| --- | --- | --- | --- | --- | --- | --- |
|  | β (95%CI) | P | β (95%CI) | P | β (95%CI) | P |
| **Negative control outcome (N=1109)** | | | | | | |
| healthy | ref |  | ref |  | ref |  |
| intermediate | 48.2(-0.4, 96.9) | 0.055 | 27.9(-8.7, 64.6) | 0.139 | 30.8(-13.4, 75.1) | 0.176 |
| poor | 55.9(-8.4, 120.2) | 0.092 | 36.8(-19.2, 92.8) | 0.201 | 13.6(-44.3, 71.4) | 0.647 |

CI, confidence intervals.

Model 1: no covariates were adjusted.

Model 2: age, sex, race/ethnicity and education level were adjusted.

Model 3: age, sex, race, education, BMI, waist circumference, smoking status, alcohol drinking status and sedentary time were adjusted.

**Table S5 Baseline characteristics of participants within included and excluded ( Age ≥20) group**

|  | **Excluded participants (Age ≥20)** | **Included participants** | **P value** | **SMD** |
| --- | --- | --- | --- | --- |
| N | 22821 | 11949 |  |  |
| Age (median [IQR]), years | 49.00 [34.00, 64.00] | 50.00 [36.00, 64.00] | 0.003 | 0.031 |
| Gender (%) |  |  | <0.001 | 0.060 |
| male | 10833 (47.5) | 6030 (50.5) |  |  |
| female | 11988 (52.5) | 5919 (49.5) |  |  |
| Race/ethnicity (%) |  |  | <0.001 | 0.091 |
| Mexican American | 3372 (14.8) | 1838 (15.4) |  |  |
| Other Hispanic | 2340 (10.3) | 1329 (11.1) |  |  |
| Non-Hispanic White | 9031 (39.6) | 5017 (42.0) |  |  |
| Non-Hispanic Black | 5181 (22.7) | 2296 (19.2) |  |  |
| Other Race | 2897 (12.7) | 1469 (12.3) |  |  |
| BMI (median [IQR]), kg/m^2^ | 27.50 [23.30, 32.30] | 28.00 [24.30, 32.50] | <0.001 | 0.236 |
| Waist (median [IQR]), cm | 94.60 [80.50, 107.00] | 98.20 [88.00, 109.00] | <0.001 | 0.501 |
| Sleep pattern (%) |  |  | 0.476 | 0.014 |
| healthy | 8255 (36.4%) | 4365 (36.6%) |  |  |
| intermediate | 8881 (39.1%) | 4596 (38.5%) |  |  |
| poor | 5553 (24.5%) | 2973 (24.9%) |  |  |
| Smoking status (%) |  |  | <0.001 | 0.046 |
| former | 5267 (23.1) | 2978 (24.9%) |  |  |
| never | 12834 (56.3) | 6640 (55.6%) |  |  |
| now | 4711 (20.7) | 2330 (19.5%) |  |  |
| Sleep duration (%) |  |  | <0.001 | 0.051 |
| 7-9 hours | 13496 (59.4) | 7237 (60.6) |  |  |
| <7 hours | 7784 (34.3) | 4092 (34.2) |  |  |
| >9 hours | 1446 ( 6.4) | 620 ( 5.2) |  |  |
| Sleep disorder (%) |  |  | 0.381 | 0.021 |
| no | 16446 (72.1) | 8545 (71.5) |  |  |
| yes | 6375 (27.9) | 3404 (28.5) |  |  |
| Trouble sleeping (%) |  |  | 0.054 | 0.032 |
| no | 17107 (75.0) | 8819 (73.8) |  |  |
| yes | 5714 (25.0) | 3130 (26.2) |  |  |
| Hypertension (%) |  |  | 0.015 | 0.028 |
| no | 10808 (47.4) | 5494 (46.0) |  |  |
| yes | 12013 (52.6) | 6455 (54.0) |  |  |
| Diabetes (%) |  |  | <0.001 | 0.074 |
| no | 18891 (82.8) | 9546 (79.9) |  |  |
| yes | 3930 (17.2) | 2403 (20.1) |  |  |
| CKD (%) |  |  | <0.001 | 0.056 |
| no | 19982 (87.6) | 10233 (85.6) |  |  |
| yes | 2839 (12.4) | 1716 (14.4) |  |  |
| CVD (%) |  |  | 0.328 | 0.011 |
| no | 20242 (88.7) | 10641 (89.1) |  |  |
| yes | 2579 (11.3) | 1308 (10.9) |  |  |
| SII Quartile (%) |  |  | <0.001 | 0.134 |
| Q1 ( <311.17) | 3344 (16.9) | 2450 (20.5) |  |  |
| Q2 (311.17–434.75) | 4639 (23.5) | 3121 (26.1) |  |  |
| Q3 (434.75–619.88) | 5534 (28.0) | 3150 (26.4) |  |  |
| Q4 ( >619.88) | 6238 (31.6) | 3228 (27.0) |  |  |
| SIRI Quartile (%) |  |  | <0.001 | 0.189 |
| Q1 ( <0.64) | 2902 (14.7) | 2350 (19.7) |  |  |
| Q2 (0.64–0.94) | 4641 (23.5) | 3216 (26.9) |  |  |
| Q3 (0.94–1.40) | 5700 (28.9) | 3255 (27.2) |  |  |
| Q4 ( >1.40) | 6512 (33.0) | 3128 (26.2) |  |  |

Median (IQR) for continuous variables, Number (%) for categorical variables.

IQR, interquartile range; BMI, body mass index; CKD, chronic kidney disease; CVD, cardiovascular disease; SII, systemic immune inflammation index; SIRI, systemic immune response index.

**Table S8 Associations between Sleep Patterns and Severity of CKM Syndrome: Results from Ordinal Logistic Regression**

|  | **Model 1** | | **Model 2** | | **Model 3** | |
| --- | --- | --- | --- | --- | --- | --- |
|  | **OR (95%CI)** | **P** | **OR (95%CI)** | **P** | **OR (95%CI)** | **P** |
| Cardiovascular-kidney-metabolic syndrome | | | | | | |
| healthy | ref |  | ref |  | ref |  |
| intermediate | 1.33(1.21,1.47) | <0.001 | 1.23(1.11,1.37) | <0.001 | 1.21(1.08,1.35) | <0.001 |
| poor | 2.57(2.27,2.90) | <0.001 | 2.31(2.00,2.68) | <0.001 | 1.64(1.37,1.95） | <0.001 |

OR, odds ratio; CI, confidence intervals.

Model 1: no covariates were adjusted.

Model 2: age, sex, race/ethnicity and education level were adjusted.

Model 3: age, sex, race, education, BMI, waist circumference, smoking status, alcohol drinking status and sedentary time were adjusted.

**Figure S1**


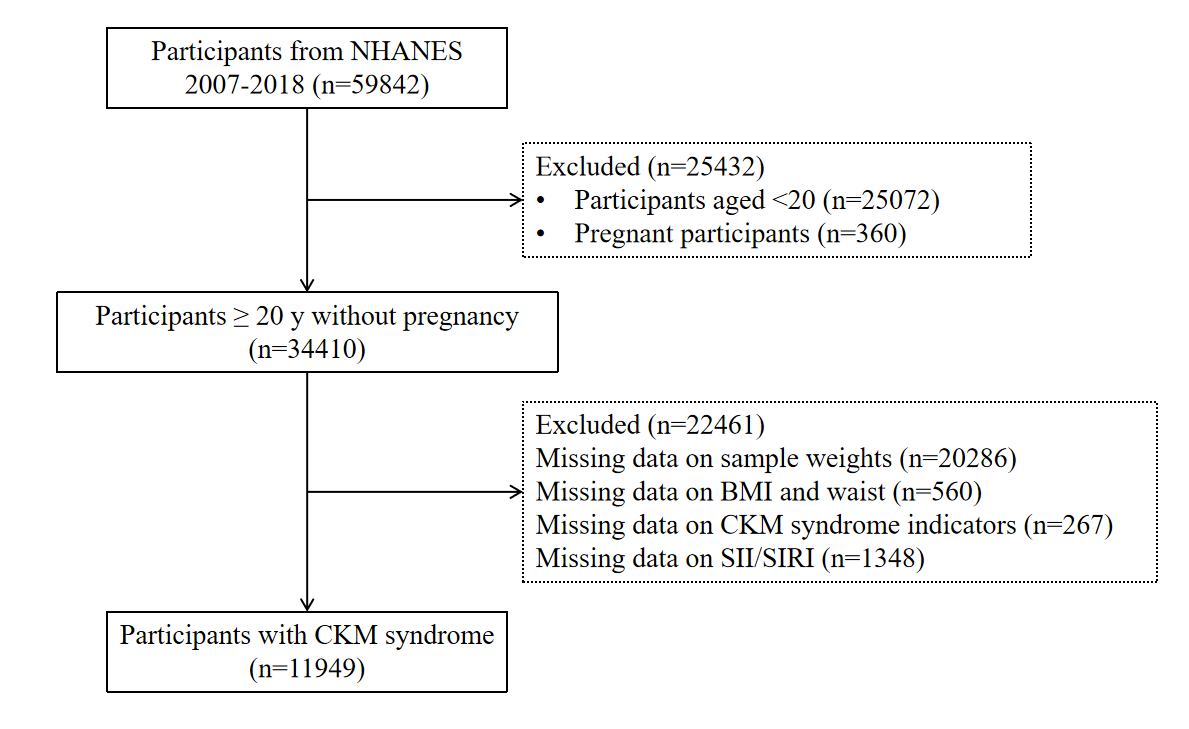


**Flowchart showing the selection of the studied participants.**

**Figure S2**


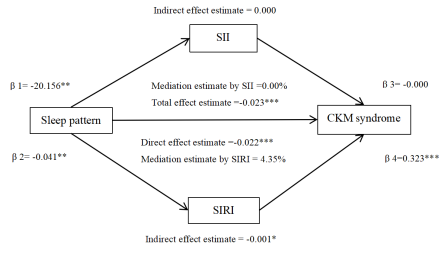

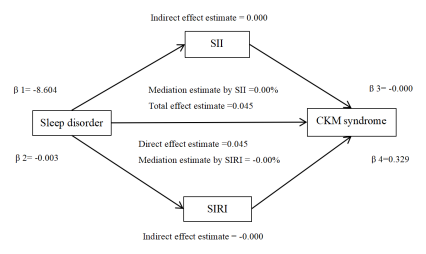

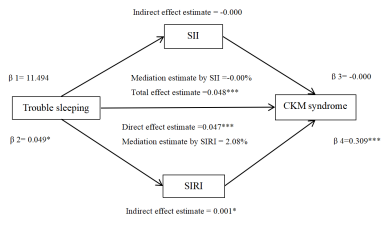


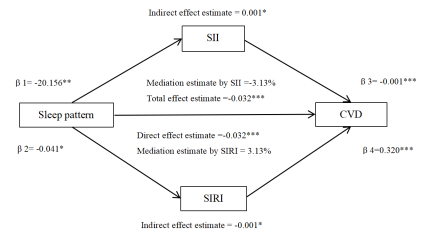

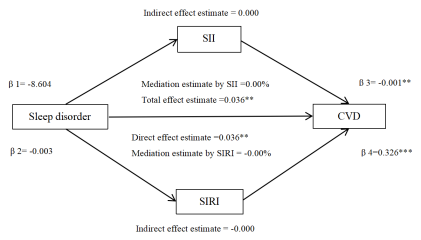

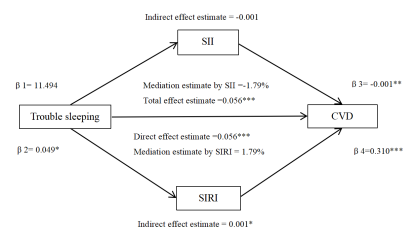


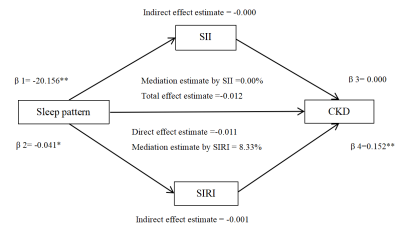

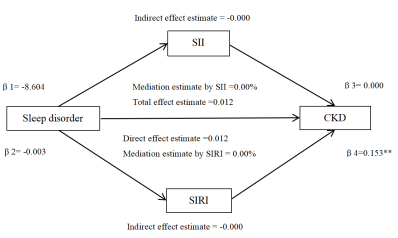

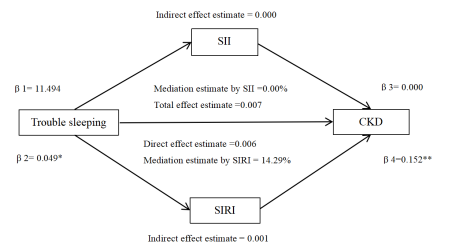


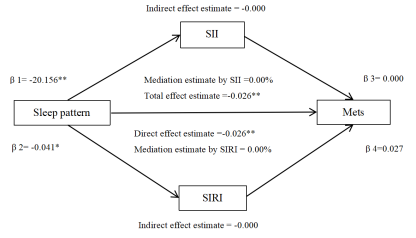

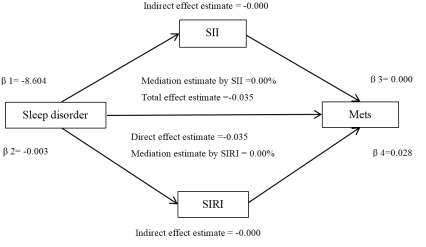

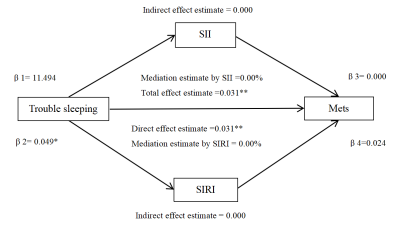


**Mediation analysis of sleep pattern, sleep disorder and trouble sleeping with CKM syndrome, CVD, CKD, and Mets.**

Mediation analysis was adjusted for age, sex, race, educational level, BMI, Waist, smoking status, alcohol drinking, sedentary time. Abbreviations: CVD, cardiovascular disease; CKD, chronic kidney disease; Mets, metabolic syndrome.
